# Knowledge and Practice in Cochlear Re-Implantation in the UK: A Survey for Audiologists

**DOI:** 10.3390/audiolres14040055

**Published:** 2024-07-17

**Authors:** Muhammed Ayas, Rosalyn Parker, David Muir, Jameel Muzaffar

**Affiliations:** 1 College of Health Sciences, University of Sharjah, Sharjah P.O. Box 27272, United Arab Emirates; 2 Cambridge Hearing Group, University of Cambridge, Cambridge CB2 1TN, UK; 3 Emmeline Centre, Cambridge University Hospitals NHS Foundation Trust, Cambridge CB2 0QQ, UK; 4 Northern Medical Physics and Clinical Engineering, Newcastle upon Tyne Hospitals NHS Foundation Trust, Newcastle NE4 5NR, UK; 5 Ear, Nose and Throat Surgery, University Hospitals Birmingham NHS Foundation Trust, Birmingham B13 8QY, UK

**Keywords:** cochlear implants, re-implantation, revision, audiologists, knowledge, practices, programming

## Abstract

Background: Cochlear implantation (CI) has proven to be a highly effective method for rehabilitating individuals with severe to profound hearing loss. However, challenges persist, particularly in cases where CI failure necessitates re-implantation. This study aims to address the gap in understanding the knowledge and practices of audiologists in the UK regarding cochlear re-implantation through a comprehensive questionnaire survey. Methods: A bespoke questionnaire was distributed to audiologists working with CI across the UK. The survey, which included multiple-choice items, open-text responses, and visual analogue scales, was made accessible via an online link shared through professional bodies, email groups, and social media platforms. Results: The survey received 27 responses, predominantly from female audiologists (71.4%), with significant representation from London (28.6%) and the East of England (21.4%). A majority of respondents had over 16 years of CI experience (35.7%) and held a master’s degree (60.7%). Key reasons for CI re-implantation included electrode failure (82.1%) and hermetic seal failure (60.7%). While respondents showed strong confidence in counselling (88.8%) and managing re-implanted devices (84.6%), there was a noted variation in opinions regarding the need for additional training in intraoperative measures. Conclusion: This survey highlights the current practices and training needs of UK audiologists in CI re-implantation. This underscores the importance of targeted training to fill knowledge gaps and improve clinical care during CI re-implantation, ultimately enhancing outcomes for both audiologists and CI recipients.

## 1. Introduction

Cochlear implants (CIs) have revolutionised hearing rehabilitation options for individuals with severe to profound hearing loss, offering life-changing access to sound. By bypassing damaged parts of the inner ear, these surgically implanted devices directly stimulate the auditory nerve, enabling recipients to perceive sound [1,2]. CIs not only enhance communication but also foster social integration, educational attainment, and emotional well-being, significantly impacting the quality of life for recipients worldwide [3]. Over the past three decades, CIs have become a pivotal intervention for individuals with severe to profound hearing loss. Despite their overall success, a subset of recipients encounters challenges such as implant device failure or infections, necessitating re-implantation [4].

The potential for implant failure and complications requiring CI re-implantation remains a concern. Re-implantation involves the removal and replacement of the malfunctioning device, necessitating a critical understanding of the detection of malfunctioning devices, management/mitigation, and subsequent rehabilitation [5,6]. As an evolving field, CI re-implantation plays a crucial role in ensuring sustained auditory benefits for this group of recipients. Several studies have reported significant speech perception improvements post-re-implantation, underscoring the importance of timely intervention [7,8,9].

Re-implantation can be a source of significant stress for patients who may already be dealing with the disappointment of device failure. Exploring the psychological impacts and the role of audiologists in managing patient expectations and providing emotional support is crucial. Discussing strategies to support patients through the re-implantation process can highlight the holistic role of audiologists in patient care. Despite recipients expressing apprehension towards surgery, CI re-implantation has been shown to positively impact the quality of life and psychosocial well-being of recipients [10]. These findings highlight the enduring benefits of CI re-implantation and stress the importance of long-term follow-up and rehabilitation for optimal outcomes.

The procedure of CI re-implantation is potentially complex and fraught with unique challenges that differ from initial implantation. A key concern is the risk of cochlear damage during the removal of the faulty device, which can complicate the insertion of the new implant and potentially affect auditory outcomes. Additionally, the presence of scar tissue from the initial surgery can pose technical difficulties for electrode array insertion and increase the risk of complications [5,6].

Audiologists play a pivotal role in the CI re-implantation process, from pre-surgical assessments to post-surgical rehabilitation. Their expertise is crucial in diagnosing device failures, counselling patients, and adjusting the programming of re-implanted devices. Given the technical and clinical complexities involved, there is a clear need for targeted training and continuous professional development to ensure audiologists are well-equipped to manage re-implantation cases effectively.

Despite the critical role of audiologists, there is significant variability in practices and training related to CI re-implantation. This variability can impact the quality of care and outcomes for patients undergoing re-implantation. For example, differences in the techniques used for device explantation, the criteria for deciding on re-implantation, and the protocols for post-operative care can lead to inconsistent results [5,6]. Addressing these inconsistencies through standardised protocols and targeted training programs provides a route to improve patient outcomes.

Recent advancements in surgical techniques and implant technology have improved the outcomes of CI re-implantation. Innovations such as minimally invasive surgical approaches and enhanced electrode designs have reduced the risks associated with re-implantation and improved auditory performance post-surgery [7,8,9]. However, these advancements also necessitate ongoing education for audiologists to stay abreast of new developments and integrate them into clinical practice.

Despite its importance, the intricacies surrounding CI re-implantation remain largely unexplored in the current literature, particularly regarding the knowledge and practices employed by audiologists. This national survey aims to comprehensively investigate the landscape of CI re-implantation in the United Kingdom (UK), with a specific focus on the roles and approaches of CI audiologists in implant failure detection, re-implantation processes, and management strategies.

The primary objective of this survey is to assess audiologists’ knowledge concerning implant failure detection, re-implantation processes, and management strategies. Additionally, the survey seeks to explore routine practices employed in addressing potential re-implantation cases in the UK, thereby providing insights into the current state of clinical practice and identifying areas for improvement. By shedding light on these aspects, this study aims to contribute to the optimization of re-implantation outcomes and enhance the overall quality of care for CI recipients.

## 2. Materials and Methods

### 2.1. Study Design 

A cross-sectional design was employed, utilising both quantitative and qualitative methodology. A bespoke questionnaire was distributed online to audiologists working with CI across the UK. Recruitment was conducted via social media and audiology professional and scientific associations such as the British Society of Audiology (BSA), British Academy of Audiology (BAA), British Cochlear Implant Group (BCIG), and the National Institute for Health and Care Research (NIHR) National Specialty Group. The data collection period for the study was from 14 December 2023 to 24 January 2024. The study was conducted in accordance with the Declaration of Helsinki. Participation was voluntary, with informed consent obtained online. Ethical approval was deemed unnecessary as data collection and analysis were considered a national service evaluation, confirmed by consultations with the Health Research Authority (HRA) and the Newcastle upon Tyne Hospitals Joint Research Office (NJRO).

### 2.2. Participants and Sample

Participants included CI audiologists involved in the care of both adult and paediatric patients based in various UK departments. The estimated population of CI audiologists in the UK is approximately 100. A total of 27 responses were obtained, providing an 80% chance of detecting correlations of ±0.223 at *p* ≤ 0.05, based on Cohen’s power analysis equation [11]. 

### 2.3. Questionnaire Development

The questionnaire was formulated after a comprehensive literature review on CI re-implantation. It included 29 questions divided into three sections:

1. Demographics: Five questions on age, sex, years of experience, and educational qualifications.
2. Knowledge: Nine questions assessing knowledge levels.
3. Practices: Fifteen questions exploring CI re-implantation practices and experience.

The questionnaire featured multiple-choice items, open-text responses, and visual analogue scales (VASs) to capture detailed insights (Supplementary Material).

### 2.4. Validation and Reliability

A panel of five experienced CI audiologists from the Emmeline Centre for Hearing Implants, Cambridge, provided feedback on the questionnaire, leading to refinements. Eight CI audiologists then assessed the questionnaire for clarity and feasibility. The internal consistency was measured using Cronbach’s alpha (0.79–0.88), indicating good validity. Reliability was assessed by administering the questionnaire twice to the same group, with Kappa scores (0.66 to 0.74) indicating substantial agreement. These pilot samples were excluded from the final analysis.

### 2.5. Statistical Analysis 

Descriptive statistics were used to analyse the data, which were expressed as percentages.

## 3. Results

### 3.1. Demographic Characteristics of Participants

A total of 27 respondents completed the survey. The majority of respondents were female (71.4%), with two participants preferring not to disclose their gender. In terms of clinical practice locations, the highest representation came from London (28.6%) and the East of England (21.4%). Table 1 summarises the demographic features of the participants.

Participants displayed diverse levels of experience with CI. A significant portion (35.7%) reported having more than 16 years of experience, followed by those with 6 to 10 years of experience (28.6%). Regarding educational qualifications, the majority (60.7%) held a master’s degree or had completed the scientific training programme (STP) in audiology. A smaller proportion held a bachelor’s degree (14.1%), while higher proportions held a Doctorate of Audiology (10.7%) or a Doctor of Philosophy (7.1%). Additionally, one respondent specified other educational qualifications, such as the British Association of Audiology Technicians (BAAT).

### 3.2. Knowledge of CI Re-Implantation 

Approximately half of the CI centres captured by the survey performed more than 100 CI implants annually (53.6%), with 57.7% of respondents supporting both paediatric and adult referrals. All four CI manufacturers (Advanced Bionics, Cochlear, MED-EL and Oticon Medical) available to the National Health Service (NHS) UK were represented among the surveyed clinicians. Referral rates for re-implantation varied, with 29.6% and 39.3% of respondents referring one to five adult or paediatric patients, respectively, per year. Some participants reported higher referral rates of 6–10 per year (18.5% for adults, 14.4% for paediatric patients). A significant proportion of respondents had not made a referral for re-implantation in the past 12 months (37.0% for adults, 32.2% for paediatric patients).

**Figure 1:**
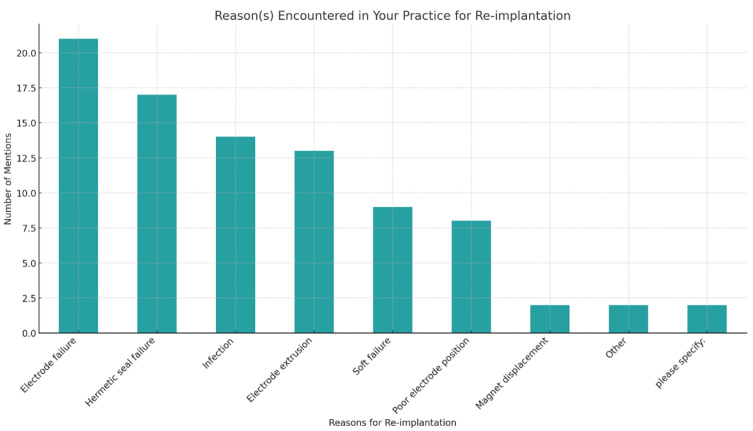
Reasons for cochlear re-implantation.

Decision making regarding CI re-implantation was influenced by various factors. The most prevalent reasons for CI re-implantation were electrode failure (82.1%) and hermetic seal failure (60.7%). Other factors included infection (46.4%) and electrode extrusion (42.9%) (Figure 1). Factors cited as barriers towards CI re-implantation included patient expectations (31.8%), cognitive decline (31.8), suitability for surgery (medical/health) (31.8%), anatomical issues (22.7%), age (22.8%), and patient choice (18.1%).

Speech testing practices included the Bamford–Kowal–Bench (BKB) Sentence Test, Arthur Boothroyd (AB) Word Lists, and McCormick Test to assess functional performance and adjust programming in patients with suspected device failure. Over half of the respondents (53.6%) were extremely familiar with making CI programming changes, while 35.7% were very familiar.

Consideration for contralateral re-implantation during revision surgery varied, with 43.5% sometimes considering it in paediatric cases, while 26.1% never did. Regarding patient-reported difficulties, 57.1% somewhat agreed that significant challenges in daily activities warranted re-implantation, and 50.0% strongly agreed that integrity testing of CI (objectively measures the voltages produced by the biphasic current pulses at the electrode array) is mandatory. Additionally, 42.9% were neutral about whether the same electrode design and manufacturer should be used during re-implantation.

Respondents strongly agreed (53.6%) or somewhat agreed (42.9%) that they could recognise clinical signs indicating the need for CI re-implantation. A significant majority (88.8%) felt confident in their ability to counsel patients requiring CI re-implantation, with 51.9% strongly agreeing and 37.0% somewhat agreeing. Additionally, 84.6% strongly agreed that they had the necessary knowledge to programme a re-implanted CI device.

### 3.3. Clinical Practices in CI Re-Implantation 

**Figure 2:**
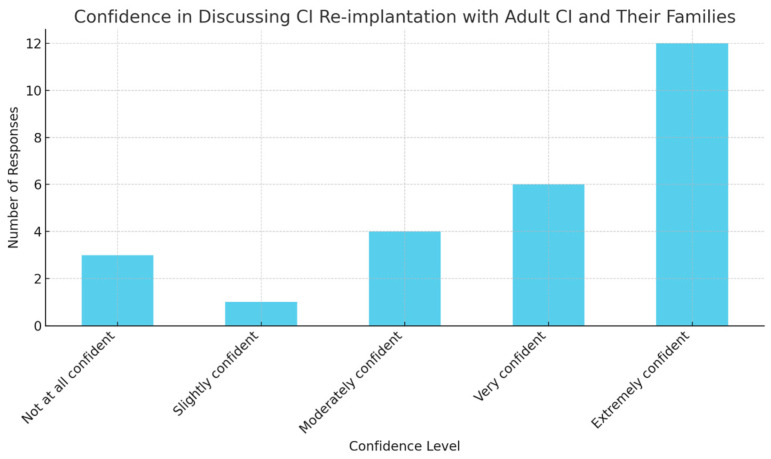
Participants’ confidence in discussing re-implantation.

Most respondents (33.3% for adults, 29.3% for paediatric patients) would discuss CI re-implantation at the time of integrity testing, while a significant portion (71.4%) expressed being “extremely confident” in discussing the need for CI re-implantation with adult CI users and their families, while for paediatric CI patients, confidence levels varied, with 41.7% feeling “very confident” and 41.7% feeling “extremely confident” (Figure 2).

Half of the respondents (50.0%) strongly agreed that they possess sufficient training and knowledge to conduct intraoperative measures during re-implantation surgery, with an additional 25.0% somewhat agreeing. However, 17.8% either somewhat disagreed or strongly disagreed with their training and preparedness for intraoperative procedures.

Respondents expressed varying levels of agreement regarding the need for more training in CI re-implantation. While 32.1% strongly disagreed and 25.0% somewhat disagreed, 25.0% were neutral. Specific areas for training highlighted by 28.6% of respondents included interpreting EFI (Electrical-Field Imaging) results, counselling patients, and understanding patterns of failure and outcomes.

## 4. Discussion

In this national survey, we conducted a comprehensive investigation into the landscape of CI re-implantation in the UK, with a specific focus on the roles and approaches of CI audiologists.

### 4.1. Knowledge of CI Re-Implantation 

Understanding clinicians’ knowledge about detecting implant failures, performing re-implantation, and managing related issues varied among participants. While most practitioners demonstrated a comprehensive understanding, others displayed notable gaps, particularly in areas such as intraoperative measures during re-implant surgery and the programming of re-implanted CI devices. These findings underscore the necessity for ongoing education and training initiatives, as partial insertions or extrusion of electrodes can occur during re-implantation. Knowledge of measuring and detecting such issues during intraoperative testing could mitigate the deactivation of electrodes during switch-on [12,13].

Continuous professional development programs tailored to address specific knowledge deficits could significantly enhance audiologists’ competency in providing comprehensive care for CI recipients. Furthermore, collaborative efforts among implant centres to share best practices and foster knowledge exchange could contribute to standardising CI protocols and improving patient outcomes across the board [14]. Additionally, the implementation of standardised protocols and guidelines for re-implantation procedures could serve as a valuable resource for audiologists, ensuring consistency and adherence to best practices.

### 4.2. Factors Influencing CI Re-Implantation Practice

This work identified a wide range of approaches employed by audiologists in addressing CI re-implantation cases. While some common practices were observed, such as the use of integrity tests and discussions with patients regarding the need for re-implantation, there were also notable variations in clinical protocols and decision-making processes. These variations may be influenced by factors such as years of experience, training background, and institutional protocols. However, the implications of these variations on patient outcomes warrant further investigation.

Experienced audiologists tended to demonstrate greater confidence and proficiency in addressing re-implantation cases, whereas less experienced audiologists may require additional support and training to enhance their skills in this area. Additionally, institutional protocols and resources may also impact audiologists’ practices, highlighting the importance of standardising clinical guidelines and providing access to educational resources.

### 4.3. Knowledge and Practice on CI Functional Performance

Clinician proficiency in assessing functional performance and making programming adjustments in patients with suspected device failure emerged as a key determinant of successful CI management. The majority of respondents reported high levels of familiarity with these tasks, highlighting the significance of training and adherence to best-practice guidelines. Effective programming is essential for optimising patient outcomes by addressing device-related issues such as speech perception, comfort, and audibility [15]. Moreover, proficiency in troubleshooting device issues and adapting programming strategies to individual patient needs is instrumental in mitigating the impact of device failures and minimising the impact on speech perception during the re-implantation process.

The choice of speech tests for decision support in re-implantation varied across centres, reflecting the needs and preferences of clinicians and patients. Standardised tests such as the BKB Sentence Test and AB Word Lists were commonly employed, offering valuable insights into patients’ speech perception abilities across different listening conditions. However, the selection of speech tests may also be influenced by factors such as patient age, language proficiency, and cognitive abilities [16].

### 4.4. Other Trends and Vulnerability of CI Failure 

The comments shared in the free-text responses provided important viewpoints on various aspects of CI re-implantation. Concerns regarding the vulnerability of certain CI devices highlight the importance of ongoing observation and careful device selection and management. Participants also raised apprehensions about the re-implantation process, especially in cases where patients transition from one manufacturer’s device to another, underscoring the need for individualized approaches to ensure optimal outcomes.

Furthermore, the emphasis placed on counselling and discussion aligns with the literature on patient-centred care and shared decision making [17]. Effective communication and managing patient expectations are critical in navigating the complexities of re-implantation, facilitating informed choices, and fostering trust between healthcare professionals and patients [18].

The development of educational resources, such as toolkits for audiologists, represents a proactive step towards enhancing clinical practice and empowering healthcare professionals with up-to-date information and strategies. These resources can boost the knowledge base of audiologists and facilitate meaningful discussions with patients, enabling them to make informed decisions about their care.

Participants also highlighted the financial impact of manufacturer issues causing CI failures, stressing the need for better support and teamwork to prioritise patient care despite workload challenges.

### 4.5. Implications for Clinical Practice

The findings of this national survey have several implications for clinical practice and patient care. Addressing knowledge gaps and standardising clinical practices are essential steps towards improving the quality of care provided to CI recipients undergoing re-implantation. 

A critical aspect revealed by the survey is the variation in knowledge and practices among CI audiologists regarding re-implantation. To bridge these gaps, it is essential to conduct comprehensive needs assessments regularly. Such assessments would identify specific knowledge deficits and areas where audiologists feel less confident, thereby enabling the creation of structured educational programs tailored to address these gaps. Utilizing advanced training methods, such as simulation-based training and online training modules, can provide opportunities for audiologists to practice complex procedures and troubleshooting without risk to patients, especially as re-implantation thankfully remains relatively uncommon.

Standardisation of clinical protocols across centres would be facilitated by the development and dissemination of national guidelines for CI re-implantation. Such guidelines would include best practices, protocols, and procedural checklists. The development of the UK National Registry for Hearing Implants (RNHI), a centralised database, will allow for the tracking and measurement of cases of re-implantation and the sharing of data surrounding this for trend analysis and benchmarking against national standards. Encouraging regular peer review and audits of CI re-implantation practices within and between centres will ensure adherence to guidelines and foster a culture of accountability and continuous improvement.

Ongoing education and training are vital to keep audiologists updated on the latest advancements and best practices in CI re-implantation. Hands-on workshops at national conferences and CI group meetings, covering practical skills, case studies, and interactive sessions with experts in the field, are likely to be well received. Developing comprehensive online teaching modules in collaboration with experienced CI clinicians will provide accessible resources covering various aspects of CI re-implantation. 

Ensuring that the re-implantation process is patient-centred is crucial for enhancing patient satisfaction and outcomes. Developing context-appropriate educational materials for patients and their families, explaining the re-implantation process, potential outcomes, and postoperative care, will further support patient-centred care. Implementing robust feedback mechanisms to gather patient perspectives on the re-implantation process and using this feedback to continuously improve clinical practices would support ongoing iterative improvements.

To ensure the effectiveness of these initiatives, continuous monitoring and evaluation are necessary. Conducting regular surveys to assess the impact of educational programs and standardised practices on audiologists’ knowledge and patient outcomes will provide valuable insights. Defining and tracking key outcome metrics related to CI re-implantation, such as device failure rates, patient satisfaction, and functional performance post-re-implantation, will allow for the ongoing assessment of progress.

### 4.6. Limitations

It is important to acknowledge the limitations of this study, which include limited participation from some parts of the UK and potential biases in survey responses. Notably, there was no representation from Northern Ireland in the survey. Additionally, since the survey focused solely on CI audiologists in the UK, the generalisability of the findings to other contexts, such as views and practices from ENT professionals, may be limited. Future research should aim to overcome these limitations by incorporating larger and more diverse samples and employing mixed-methods approaches to gain a comprehensive understanding of CI re-implantation practices.

### 4.7. Future Research Directions

Future research should focus on longitudinal studies to track the long-term outcomes of CI re-implantation and identify factors that contribute to successful re-implantation. There is also a need for studies that explore the psychological impact of re-implantation on patients and their families, as well as the development of interventions to support them through the process. Repetition of this survey, or an expanded version, in the UK would allow for the tracking of changes in confidence and approach over time. Similar studies in other countries would allow for international comparisons, which may facilitate the spread of interventions demonstrated to improve audiologist confidence. 

## 5. Conclusions

This national survey investigated UK CI audiologists’ practices in CI re-implantation to enhance care quality. It uncovers clinical trends, training needs, and knowledge gaps across centres, fostering knowledge sharing. The survey aims to outline prevalent practices, inform targeted training, and enhance care standards, ultimately improving clinical care during CI re-implantation for both audiologists and CI recipients.

## Figures and Tables

**Table 1:** Demographic characteristics of the participants.

Demographic Characteristic	Count	Percentage
Sex		
Female	20	71.4%
Male	5	17.9%
Prefer not to say	2	7.4%
Clinical Practice Location		
East of England	6	22.2%
London	8	29.6%
South East	2	7.4%
West Midlands	2	7.4%
Yorkshire and The Humber	2	7.4%
North West England	1	3.7%
Scotland	1	3.7%
South West	1	3.6%
Wales	2	7.4%
North East England	1	3.7%
East Midlands	1	3.7%
Years of CI Experience		
0–5 years	3	11.1%
6–10 years	8	28.6%
11–15 years	6	21.4%
≥16 years	10	35.7%
Education Level		
Bachelor’s degree	4	14.1%
Master’s degree/STP	17	60.7%
Doctorate of Audiology	3	10.7%
Doctor of Philosophy	2	7.1%
Other, please specify	1	3.7%

## Data Availability

The datasets used and analysed in the current study can be obtained from the corresponding author, subject to reasonable request.

## Supplementary Materials

The following supporting information can be downloaded at: https://www.mdpi.com/article/10.3390/audiolres14040055/s1, Questionnaire: Knowledge and practice in cochlear re-implantation—A survey for Audiologists
