# Supplementary material for: Knowledge and Practice in Cochlear Re-Implantation in the UK: A Survey for Audiologists
Source: Audiol Res. 2024 Jul 17;14(4):649–58. doi: 10.3390/audiolres14040055 (PMC11270342; doi:10.3390/audiolres14040055)
Supplement: Supplementary file 1 [file audiolres-14-00055-s001.zip › audiolres-3048247-supplementary.pdf]

Knowledge and practice in cochlear re-implantation - A survey for Audiologists

Gender

- Man
- Woman
- Non-binary
- Other
- Prefer not to say

1. **What population of CI patients do you serve?**
   - Paediatrics being 0-18 years old
   - Adults being 18 years old or more
   - Both paediatrics and adults

2. **In which part of the UK is your clinical practice?**
   - North East England
   - North West England
   - East of England
   - London
   - South East
   - South West
   - Yorkshire and The Humber
   - East Midlands
   - West Midlands
   - Scotland
   - Wales
   - Northern Ireland

3. **How long have you been working with CIs?**
   - 0-5 years
   - 6-10 years
   - 11-15 years
   - ≥16 years

4. **What is your highest level of education?**
   - Bachelor’s degree
   - Master's degree/STP
   - Doctorate of Audiology
   - Doctorate of Philosophy

5. **How many CIs does your centre implant per year?**
   - 0-12
   - 13-24

- 25-50
- 51-100
- >100

6. **How many adult CI patients have you referred for a CI re-implant over the last 12 months?**
   - None
   - 1 to 5
   - 6 to 10
   - More than 10

7. **How many paediatric CI patients have you referred for a CI re-implant CI over the last 12 months?**
   - None
   - 1 to 5
   - 6 to 10
   - More than 10

8. **What is the most common reason for re -implantation (please select one):**
   - Electrode failure
   - Hermetic seal failure
   - Soft failure
   - Infection
   - Magnet displacement
   - Poor electrode position
   - Electrode extrusion
   - Other (please list): ________________________

9. **Which factors, if any, make a patient not suitable for a CI re-implantation? Select all that apply**
   - Unrealistic expectations
   - Cognitive decline
   - Age
   - Never deny CI reimplantation when criteria are met

11. How familiar are you in assessing the functional performance and making programming changes in patients with suspected device failure
- Extremely familiar
- Very familiar
- Moderately familiar
- Slightly familiar
- Not at all familiar

12.Which speech tests do you use for your decision to support re-implantation? (select all that apply)

- BKB (Bamford, Kowal and Bench) Sentence test in noise
- BKB (Bamford, Kowal and Bench) Sentence test in quiet
- CUNY (City University of New York) Sentence test
- AB (Arthur Boothroyd) Word lists
- McCormick test
- Other speech tests used, please list: ___________________________
- None

13.In paediatric CI, if re-implantation is necessary for one ear due to device failure, would your clinic consider re-implant the contralateral ear at the same time?

- Always
- Often
- Sometimes
- Rarely
- Never

Please indicate your opinion on the following statement by selecting one of the options below;

14. A CI candidate should experience a significant challenge in activities of daily living to be considered for re-implantation

- Strongly agree
- Somewhat agree
- Neither agree nor disagree
- Somewhat disagree
- Strongly disagree

15. An integrity test is mandatory to consider a patient for re-implantation

- Strongly agree
- Somewhat agree
- Neither agree nor disagree
- Somewhat disagree
- Strongly disagree

16. If a patient undergoes re-implantation should the patient be implanted with same electrode design and manufacturer?

- Strongly agree
- Somewhat agree
- Neither agree nor disagree
- Somewhat disagree
- Strongly disagree

  If you disagree or strongly disagree Please comment

17. At what point of the CI pathway would you discuss the need for re-implantation with adult CI patients

- At the time of integrity testing
- Poor functional performance
- If the patient requests
- Other (Please specify)……

18. At what point of the CI pathway would you discuss the need for re-implantation with paediatric CI patients
- At the time of integrity testing
- Poor functional performance
- If the parent requests
- Others (Please specify) ……

19. How confident are you in discussing the need for CI re-implantation with the adult CI and their families?
- Extremely confident
- Very confident
- Moderately confident
- Slightly confident
- Not at all confident

20. How confident are you in discussing the need for CI re-implantation with the paediatric CI and their families?
- Extremely confident
- Very confident
- Moderately confident
- Slightly confident
- Not at all confident

21. I have adequate training and knowledge to recognize the clinical signs and need for CI re-implantation
- Strongly agree
- Somewhat agree
- Neither agree nor disagree
- Somewhat disagree
- Strongly disagree

22. I have adequate training and knowledge on how to counsel a patient who requires re-implantation
- Strongly agree
- Somewhat agree
- Neither agree nor disagree
- Somewhat disagree

- Strongly disagree

23. I have adequate training and knowledge on how to do intraoperative measures during the re-implant surgery
- Strongly agree
- Somewhat agree
- Neither agree nor disagree
- Somewhat disagree
- Strongly disagree

24. I have adequate training and knowledge on how to program a re-implanted CI device
- Strongly agree
- Somewhat agree
- Neither agree nor disagree
- Somewhat disagree
- Strongly disagree

25. I feel I need more training in general to deal with CI re-implantation
- Strongly agree
- Somewhat agree
- Neither agree nor disagree
- Somewhat disagree
- Strongly disagree

26. Please describe in detail the areas which you feel training is required…….

27. Any others comments or concerns related to CI re-implantation, please specify in detail…..
